# Impact of Nutritional Status on Caregiver Burden of Elderly Outpatients. A Cross-Sectional Study

**DOI:** 10.3390/nu11020281

**Published:** 2019-01-28

**Authors:** Claudio Tana, Fulvio Lauretani, Andrea Ticinesi, Luciano Gionti, Antonio Nouvenne, Beatrice Prati, Tiziana Meschi, Marcello Maggio

**Affiliations:** 1 Internal Medicine and Critical Subacute Care Unit, Geriatric-Rehabilitation Department, Azienda Ospedaliero-Universitaria di Parma, 43126 Parma, Italy; andrea.ticinesi@unipr.it (A.T.); ANouvenne@ao.pr.it (A.N.); bprati@ao.pr.it (B.P.); tiziana.meschi@unipr.it (T.M.); 2 Geriatric Clinic Unit, Geriatric-Rehabilitation Department, Azienda Ospedaliero-Universitaria di Parma, 43126 Parma, Italy; flauretani@ao.pr.it (F.L.); marcellomaggio2001@yahoo.it (M.M.); 3 Cognitive and Motor Center Clinic, Geriatric-Rehabilitation Department, Azienda Ospedaliero-Universitaria di Parma, 43126 Parma, Italy; luciano.gionti82@gmail.com; 4 Department of Medicine and Surgery, University of Parma, 43126 Parma, Italy

**Keywords:** frailty, caregiver, dementia, malnutrition, disability

## Abstract

The assistance to older community-dwellers provided by family caregivers is crucial for the maintenance of an acceptable quality of life, especially when dementia is present. The caregiver burden may be extremely high, but few data are available on what patient domains mainly affect the caregiver. The aim of this cross-sectional study, performed in older outpatients, was to examine the impact of cognitive, physical and nutritional status of elderly community-dwellers on the caregiver burden, as evaluated by the Caregiver Burden Inventory (CBI). A group of 406 elderly outpatients (161 M, 245 F, mean age of 83.20 ± 6.40) was enrolled. A significant correlation was observed between Mini Nutritional Assessment Instrument-Short Form (MNA-SF) and CBI (*r* = −0.34; *p* < 0.001), suggesting that a poor nutritional status is significantly associated with the caregiver burden. There was also a significant correlation between CBI and Short Physical Performance Battery score (*r* = −0.29; *p* < 0.001), hand grip strength (*r* = −0.25; *p* < 0.001), Mini-Mental State Examination score (*r* = −0.39; *p* < 0.001), Geriatric Depression Scale (*r* = 0.23; *p* < 0.001), Body Mass Index (BMI) (*r* = 0.01; *p* = 0.03), Activities of Daily Living and Instrumental Activities of Daily Living (ADL/IADL) (*r* = −0.61 and −0.62, respectively; *p* < 0.001), and with the 4-m walking speed (*r* = −0.42; *p* < 0.001). In the multivariate analysis, only the relationships of the CBI (in particular the physical subcomponent) with ADL, IADL and MNA-SF remained statistically significant (β ± SE −0.89 ± 0.20, *p* < 0.001; −0.58 ± 0.15, *p* < 0.001 and −0.25 ± 0.11, *p* = 0.02, respectively). The relationship between CBI and BMI remained statistically significant only for the physical subcomponent (β ± SE 0.14 ± 0.05; *p* = 0.006). Thus, in this study, we confirmed that the impairment in the activities of daily living is associated with a significant impact on the caregiver burden, and we found also that a poor nutritional status of the older outpatient is independently more associated with the caregiver burden than cognitive and physical disability. The combined evaluation of both patients and caregivers can improve the knowledge and assistance to the elderly subjects.

## 1. Introduction

In the last decades, the improvement of quality of care and the consequent increase in life-expectancy have been associated with an increase of the elderly population, with higher demand for long-term care, especially in developed countries [1,2]. Despite a reduction in late-life mortality, there has been an increase of prevalence of cognitive disorders such as dementia, especially Alzheimer’s disease, vascular dementia, and combination of both (mixed dementia) [3,4,5]. At the same time, there has been an increase of multiple chronic conditions, and their combination, i.e., multimorbidity [6]. 

Elderly people with dementia and/or multiple chronic conditions often have a high dependency in daily activities and are more prone to develop disability [7]. Family caregivers usually provide crucial assistance to the elderly and have several responsibilities, such as supervision, drug administration, mobilization, hygiene and psychological support [7]. As disability progresses, caregivers may experience significant stress, depression, anxiety and fatigue, related both to emotional involvement and to objective burden of care, affecting multiple domains of their lives. When the same patients are admitted to the hospital, caregivers may also be forced to take job leaves, with significant social and financial consequences [8]. Several reports show that approximately 85% of patients with dementia and multiple chronic conditions are cared exclusively in their own homes, resulting in a high burden for the caregivers, who take care of patients an average time of 12 hours a day [9,10,11]. 

Several risk factors for caregiver burden leave have been identified, including low education, female sex, residence in the same house of the patient and high number of hours spent to assist [12,13,14].

Although the exposure of the caregiver risk is well known, there is a substantial lack of evidence on the association between the patient health conditions and the caregiver burden, since current data about a possible correlation are conflicting [15,16,17]. 

Most studies have focused on the relationship between patients’ cognitive status severity and caregiver burden. However, cognition is hardly ever the sole geriatric domain compromised in elderly patients with cognitive impairment or dementia. Reduced physical performance up to mobility-disability, depressive symptoms, and malnutrition frequently coexist in these patients, and each of these factors may dramatically influence the perceived caregiver burden. However, few studies have investigated the possible correlation between each of these domains, systematically assessed by comprehensive geriatric assessment (CGA), and caregiver burden. In particular, there is no information about a potential correlation between the nutritional status of elderly patients and caregiver burden. 

We hypothesized that the caregiver stress could have a defined, multifactorial and not singular etiology attributable to the multiple conditions affecting the elderly people. In this study, we examined the impact that cognitive, physical and nutritional status of elderly people have on the caregivers. 

## 2. Methods

### 2.1. Design of the Study

This was a cross-sectional, single-center, no-profit study aimed at evaluating the association between cognitive, physical performance, nutritional status of elderly outpatients and the caregiver burden. This was part of a larger project, called Traumatic Risk Identikit Parma (TRIP) study, that was aimed at assessing risk factors for falls in elderly outpatients evaluated for suspected cognitive and motoric frailty at the first visit [18,19].

### 2.2. Study Participants

Elderly outpatients consecutively referred to the Cognitive and Motor Center of the Geriatric Rehabilitation Department of Parma, Italy, were evaluated in the period September–December 2017 for a CGA due to motoric or memory complaints, as requested by their own general practitioner or after discharge from our Geriatric-Rehabilitation Department Units. Patients were included if they were aged 70 years or older. Caregivers were eligible for the study if they were aged 18 or older, were co-housing with patients and provided a minimum of 4 hours of supervision or direct care per day for at least 6 months prior to the enrollment. Caregivers were excluded if they had a significant psychiatric illness (e.g., schizophrenia or bipolar disorder) or cognitive impairment themselves. 

For the present analysis, 406 elderly outpatients aged 70 years or older (mean age 83.20 ± 6.40 (DS)), and 406 caregivers were eligible for the study. 

The TRIP study protocol was approved by the Ethics Committee of Parma Province, Italy (ID 17262 - 12/05/2017). All patients and their caregivers were informed about the study procedures and they gave their written informed consent. If patients were not able to provide their consent, a next of kin was requested to provide the consent for the participation to the study.

### 2.3. Scales

A complete clinical examination including age, gender, medical and drug history and total number of falls of the patients was performed and recorded.

All patients were examined by a trained geriatrician and a skilled nurse. The CGA consisted of the following evaluation of standardized measures: 

1. Daily Activities: The Activities of Daily Living (ADL) and Instrumental Activities of Daily Living (IADL) scales [20] were used for measuring the patients’ functional ability. ADL and IADL are widely used in clinical studies and community-based researches.

2. Physical Function: The physical performance tests included the assessment of grip strength by a hand-held dynamometer and the Short Physical Performance Battery (SPPB), i.e., hierarchical assessment of standing balance, chair-stand test and 4-m walking speed, measured with the aid of a stopwatch (4-MM) [21,22,23,24,25]. For each participant, all measures were collected during the same day and in the same order: SPPB was the first assessed, leaving 4-m gait speed assessment as its last subtest, and handgrip strength at the end of the examination. A period of 2–3 minutes of rest was granted between each test [26]. 

3. Cognitive performance: The cognitive performance was assessed using the Mini-Mental State Examination (MMSE) [27], adjusted for age and education according to the model of Magni et al [28], and the Clock Drawing Test (CDT) [29]. Depressive symptoms were assessed by using the 5-item Geriatric Depression Scale (GDS-5), which is a widely used screening instrument for evaluating the depressive symptoms in the elderly. The GDS is a self-report yes/no questionnaire that is developed specifically for older adults and that excludes somatic signs and symptoms of syndromal depression [30]. 

4. Nutritional Assessment: The nutritional status of patients was assessed by using the Mini Nutritional Assessment Instrument—Short Form (MNA-SF), which is a reliable screening tool for malnutrition in all geriatric settings. A score <7 indicates overt malnutrition, a score between 8 and 11 is indicative of a high risk of malnutrition, while a score between 12 and 14 suggests good nutritional status. The score is based on objective parameters such as BMI, weight and calf circumference, and on subjective complaints, including appetite, dismobility and psychological status [31]. 

5. Caregiver Burden Inventory: The caregiver burden was assessed by recording the caregiver burden inventory (CBI), which is a test that encompasses 24 closed questions divided into five dimensions: time-dependence, and developmental, physical, social and emotional burden. Each dimension has five items, except for the physical burden, which has four dedicated items. A score between 0 and 4 (not at all and very challenging, respectively) is provided for each item. The highest score indicates the greatest burden for caregivers [32].

### 2.4. Statistical Analyses

The statistical processing was carried out using the Statistical Analysis System (SAS) 8.2 software. Descriptive statistics such as frequencies, means and standard deviations were first obtained to explore the demographic characteristics. Age- and sex-adjusted Pearson correlation analysis was used to identify variables correlated to CBI component. Variables correlated to CBI at the univariate analyses were used in multiple linear regression models to assess the association of ADL, IADL, MMSE, CDT, GDS-5, Hand Grip strength, Gait Speed (m/s), SPPB, MNA-SF and other social-demographic variables (gender, age, weight, height, BMI, multimorbidity, and number of medications) with CBI, considering the total score of CBI and its subscores as the dependent variable. A *p*-value of less than 0.05 was considered as statistically significant.

## 3. Results

The characteristics of the patients participating to the study (*n* = 406, 161 M, 245 F) are summarized in Table 1. The mean age was 83.20 (SD: ±6.40) years old. 

The caregiver was a son or daughter in most patients (99.5%), female in 78% and male in 22% of cases. Only in 48 cases (13%) the caregiver was helped by a professional caretaker. Patients lived alone, with the caregiver being present in the house only for some hours a day, in 42 cases (10%). The caregiver was the only cohabitant in 64% of cases, while, in other cases one, usually the caregiver’s spouse, or two or more other family members were present. 

The higher value of CBI was recorded for the physical components (median 6, 95% CI 1–11). After adjusting for age and sex (Table 2), there was a significant correlation between CBI and SPPB score (*r* = −0.29; *p* < 0.001), hand grip strength (*r* = −0.25; *p* < 0.001), MMSE score (*r* = −0.39; *p* < 0.001), GDS (*r* = 0.23; *p* < 0.001), and BMI (*r* = 0.01; *p* = 0.03). There were also significant correlations between CBI and ADL/IADL (*r* = −0.61 and −0.62, respectively; both *p* < 0.001). Additionally, a strong correlation was observed with the MNA-SF (*r* = −0.34; *p* < 0.001), where a poor nutritional status had a significant impact on the caregiver burden, and with the 4-m walking speed (*r* = −0.42; *p* < 0.001). In the multivariate analyses (Table 3), the relationships between the CBI (namely, the physical subcomponent) and ADL/IADL and MNA-SF remained statistically significant (β ± SE −0.89 ± 0.20, *p* < 0.001; −0.58 ± 0.15, *p* < 0.001 and −0.25 ± 0.11, *p* = 0.02, respectively). The relationship between CBI and BMI remained statistically significant only for the physical subcomponent (β ± SE = 0.14 ± 0.05; *p* = 0.006) (Table 3). No significant correlation was observed between the emotional component of CBI and MNA-SF, motor and cognitive disability of patients.

## 4. Discussion

In this study, we found a significant correlation between the caregiver burden in its different components and the nutritional status of elderly patients, as evaluated by MNA-SF score, and with patient’s disability, as evaluated by their cognitive and physical status. We also confirmed that the reduction of independence in the activities of daily living has a significant impact on the caregiver burden. Caregiver burden can be defined as a state of psychological and/or physical discomfort associated with the need of taking care of elderly patients with severe disorders and disability [33]. 

Caregivers are important figures not only because they provide physical assistance to the patients, but also because they are responsible for the daily therapy administration and because they relief the emotional discomfort. Furthermore, they are often involved in other functions, such as household tasks and children care that may further affect their emotive and physical stress [34]. 

Previous studies have demonstrated that the patient’s disability and behavioral abnormalities are the major contributors of the caregiver burden [15,35,36]. A significant correlation between the CBI and MMSE has been demonstrated, with a consistent association with caregiver sex and length of time spent in months for taking patient’s care [37].

In a recent study, however, the strong association between the cognitive impairment of elderly patients and caregiver burden has not been confirmed, raising some questions about this relationship [16,17]. Here, we confirmed previous data about the significant influence of cognitive impairment of elderly patients on the overall caregiver burden. However, patient cognition did not seem to affect the social and emotional components of caregiver burden, but only physical aspects. We may hypothesize that the existence of a kin between caregivers and patients may reduce caregiver’s perception of their personal sacrifices in the care process of cognitively impaired persons. From this point of view, only objective elements, such as the fatigue associated with mobilization of disabled patients, may be perceived as a burden. Conversely, these data may also be interpreted as an underestimation of cognitive impairment by the caregivers, who may tend to consider as burdensome other domains of the elderly person, such as poor physical performance.

In fact, this parameter, measured as low SPPB score, reduced grip strength and slow 4-m walking speed, was significantly associated with a high caregiver burden. A lower physical performance, associated with the greater need of physical assistance to the patient, can indeed impact significantly on the caregiver status. An impaired performance of daily living activities, as evaluated by lower ADL and IADL scores, has already been associated with a high caregiver burden [38,39,40]. In our study, the finding of the inverse correlation between ADL/IADL and CBI score reinforces the evidence about the negative effects that a poor physical status and reduced patient self-sufficiency have on the caregiver assistance. In this regard, the creation of interventions aimed at improving the daily living activities could contribute to reduce the caregiver discomfort, and therefore can in turn improve the patient’s care [38].

The influence that a reduced cognitive and motoric performance has on the physical and social status of the caregivers can reflect the daily source of caregiver stress, and indicates the need for attention on measures aimed at improving the quality of caregiver work and life, taking into account that a better quality of caregiver life would reflect in a better patient’s assistance. 

Interestingly, while previous studies have demonstrated a significant impact of patient’s disability on the caregiver burden in term of emotive disturbances such as anxiety and depression [15], we did not find a significant correlation between the emotional status of the caregivers and the cognitive and motor disability of the patients at a multivariate analysis. 

A new interesting point that emerges from this study is that the nutritional status of the patients is significantly correlated with the caregiver stress. Namely, we found that the presence of components of physical frailty such as malnutrition risk, as evaluated by a low MNA-SF score, increases significantly the caregiver burden, independently from the other factors such as cognitive and physical disability. Previously, some authors aimed at identifying risk factors for caregiver burden, and they found that only a reduced performance status and hemoglobin values <12 g/dL, a proxy of poor nutritional status, were associated with an increased risk of caregiver burden in older patients with cancer at a multivariate analysis [41,42].

In a group of older patients undergoing home artificial nutrition, Villar-Taibo and colleagues found that the administration of oral supplements in combination with enteral nutrition was associated with a higher caregiver burden than total enteral nutrition [43]. Dysphagia and malnutrition were associated with higher caregiver burden also in long-term care residents [44]. These findings are not surprising in populations with a high prevalence of malnutrition or in subjects with known dysphagia. However, the present study shows that this relationship between nutritional risk and caregiver burden is present also in a less selected population of geriatric outpatients, highlighting that malnutrition is perceived as burdensome even before it is clinically evident. 

Ruller et al. found an inverse correlation between nutritional status of elderly patients and the caregiver burden (assessed with the Zarit Burden Interview), but this relationship did not reach the statistical significance (*p* = 0.08) [45]. Furthermore, this score did not explore the different components of caregiver stress, which can be influenced differently. The CBI could be more appropriate to assess the physical, social and emotive burden of caregivers, when they assist elderly outpatients in clinical practice. 

In this regard, it could be useful to integrate a nutritional risk assessment with a thorough psychosocial caregiver evaluation, because the finding of a reduced nutritional status could suggest the use of measures aimed at improving it, and of consequence the caregiver well-being and the patient’s care. Interestingly, caregivers of older persons with elevated functional dependency due to severe dementia experience themselves a higher nutritional risk [46]. In fact, many caregivers of geriatric patients may be old themselves, and experience, to a lesser degree, the same health problems [47].

A poor nutritional status may be perceived as burdensome by caregivers for several reasons. First, in Italian popular culture, a good appetite is associated with a good health status, while the “anorexia of aging” [48,49], a frequent condition of older frail subjects, is generally regarded as troublesome. Caregivers may perceive this condition as a burden because they try to force their relatives to eat more, which may be actually laborious and time-consuming. Second, malnutrition is frequently associated with poor health outcomes and a high rate of complications, including mobility-disability, low muscle strength, sarcopenia, pressure ulcers, infections, depression and worse cognitive status [50,51,52,53,54,55]. Thus, the observed correlation may depend on the presence of one or more of these conditions. Last, the presence of anorexia of aging may be associated with a significant change in the lifestyle and daily habits of patients, including a reduced capacity of sharing a meal with the family, which may be perceived as highly burdensome for caregivers [56]. 

Despite these novel findings, the study has some limitations. The cross-sectional design did not allow evaluating the relationship of causality between patients’ health condition and caregivers’ stress. A prospective design, aimed at evaluating the caregiver burden and its modification after changes of patients’ health condition, could be useful to explore the correlation between them. Another limitation was that only one scale (the CBI) was used to assess the caregiver burden. A multiscale assessment could be useful to assess globally the emotional and physical stress of caregivers in real-life. Furthermore, the socioeconomic status of both patients and caregivers was not investigated. A poor socioeconomic status could influence both the risk of malnutrition and caregiver burden independently. Finally, no demographic and clinical data on caregivers were available. As the population ages, there is in fact a consistent number of geriatric patients who are cared for by family members who are old themselves, and may experience reduced fitness, making burdensome tasks that are not perceived as such by younger people [47]. The possible presence of other burdensome tasks in the household (i.e., caring for children, cooking for multiple family members, and supervision of other subjects with functional impairment) was also not investigated. 

Despite these limitations, this study confirms several multifaceted aspects of the relationship between the geriatric community-dwelling patient’s health conditions and caregiver burden, underlining the role of nutritional status.

## 5. Conclusions

This study highlights, for the first time in an unselected group of geriatric outpatients, the correlation between their nutritional status and the burden of care experienced by their caregivers. It also confirms the significant impact of a reduced physical performance of the patients on caregiver burden. These aspects, together with the evidence of the relationship with physical and cognitive components of caregiver burden, confirms the importance of a correct identification of the caregiver sources of stress in clinical practice. Often, these components are underestimated, but a higher attention to caregivers in daily routine, along with a correct assessment of nutritional and physical status of elderly outpatients, can result in a global improvement of patients’ health condition and quality of life for the whole family. Further studies are needed to confirm this relationship, and to assess if the improvement of nutritional status of geriatric patients can have an impact in the caregiver burden. 

## Figures and Tables

**Table 1:** Characteristics of the Study population (*n* = 406).

	Mean, Median, *N*	SD, IQR, %
Age (years)	83.20	±6.40
Sex (men)	161	39.7%
CBI-Total	31.9	13.8–52.8
CBI- Physical Component	6	1–11
CBI-social component	2	0–7
CBI-emotional component	1	0–4
SPPB Total Score	5	1.5–8.0
Grip Strength (Kg)	18.50	9.0
MMSE score	20.20	±6.0
MNA-SF	10.01	±2.60
GDS	4.0	1–8
Number of Drugs		
<5	223	55%
≥5	183	45%
N. of Chronic Diseases	5.5	1–9
ADL (Katz’s scale)	4	1–6
IADL (Lawton’s scale)	1	0–5
4-m Walking Speed	0.61	0.29–0.85
BMI (Kg/m^2^)	26.90	±5.20

CBI, Caregiver Burden Inventory; SPPB, Short Physical Performance Battery; MMSE, Mini-Mental State Examination; GDS, Geriatric Depression Scale; ADL, Activities of Daily Living; IADL, Instrumental Activities of Daily Living; BMI, Body Mass Index.

**Table 2:** Age- and sex-adjusted correlation (r values) between total Caregiver Burden Inventory (CBI) score, or its subcomponents, and physical and cognitive characteristics.

	CBI-Total	CBI-Physical Component	CBI-Social Component	CBI-Emotional Component
SPPB Total Score	−0.29 (**<0.001**)	−0.34 (**<0.001**)	−0.29 (**<0.001**)	−0.13 (**0.02**)
Grip Strength (Kg)	−0.25 (**<0.001**)	−0.19 (**<0.001**)	−0.06 (0.27)	−0.05 (0.37)
MMSE score	−0.39 (**<0.001**)	−0.29 (**<0.001**)	−0.23 (**<0.001**)	−0.14 (**0.009**)
MNA−SF	−0.34 (**<0.001**)	−0.29 (**<0.001**)	−0.19 (**<0.001**)	−0.06 (0.25)
GDS	0.23 (**<0.001**)	0.20 (**<0.001**)	0.24 (**<0.001**)	0.11 (**0.048**)
Number of Drugs	0.10 (0.07)	0.09 (0.13)	0.03 (0.65)	0.03 (0.62)
BMI	0.01 (**0.03**)	0.15 (**0.006**)	0.03 (0.52)	0.01 (0.81)
4-m walking speed	−0.42 (**<0.001**)	−0.36 (**<0.001**)	−0.17 (**0.001**)	−0.12 (**0.02**)
ADL (Katz’s scale)	−0.61 (**<0.001**)	−0.53 (**<0.001**)	−0.28 (**<0.001**)	−0.19 (**<0.001**)
IADL (Lawton’s scale)	−0.62 (**<0.001**)	−0.52 (**<0.001**)	−0.29 (**<0.001**)	−0.19 (**<0.001**)

CBI, Caregiver Burden Inventory; SPPB, Short Physical Performance Battery; MMSE, Mini-Mental State Examination; GDS, Geriatric Depression Scale; ADL, Activities of Daily Living; IADL, Instrumental Activities of Daily Living; BMI, Body Mass Index. *p* values are shown within parentheses, and indicated in bold if <0.05.

**Table 3:** Multivariate regression analysis between total Caregiver Burden Inventory Score (total and subscores) and parameters of multidimensional geriatric assessment.

	Association with CBI Total Score		Association with CBI Physical Component Score		Association with CBI Social Component Score	
	β ± SE	*p* *	β ± SE	*p* *	β ± SE	*p* *
Sex (female vs. male)	8.01 ± 2.58	**0.002**	1.37 ± 0.64	**0.03**	1.87 ± 0.67	**0.006**
Age	0.13 ± 0.18	0.43	0.05 ± 0.04	0.29	0.04 ± 0.04	0.35
IADL	−2.93 ± 0.60	**<0.001**	−0.58 ± 0.15	**<0.001**	−0.29 ± 0.16	**0.01**
ADL	−4.20 ± 0.80	**<0.001**	−0.89 ± 0.20	**<0.001**	−0.39 ± 0.21	0.11
Grip strength	−0.33 ± 0.17	0.05	0.03 ± 0.04	0.49	−0.09 ± 0.04	**0.03**
SPPB score	0.30 ± 0.60	0.56	0.16 ± 0.10	0.13	0.11 ± 0.09	0.27
GDS	1.08 ± 0.76	0.16	0.27 ± 0.20	0.16	0.64 ± 0.18	**<0.001**
MMSE	−0.25 ± 0.25	0.31	0.007 ± 0.06	0.90	−0.05 ± 0.06	0.43
BMI	0.24 ± 0.20	0.23	0.14 ± 0.05	**0.006**	0.05 ± 0.48	0.30
MNA-SF	−0.86 ± 0.46	**0.04**	−0.25 ± 0.11	**0.02**	−0.07 ± 0.11	0.58

* *p* adjusted for all the considered possible confounders. *p* values < 0.05 are indicated in bold. CBI, Caregiver Burden Inventory; EAT-10, Eating Assessment Tool; SPPB, Short Physical Performance Battery; MMSE, Mini-Mental State Examination; GDS, Geriatric Depression Scale; ADL, Activities of Daily Living; IADL, Instrumental Activities of Daily Living; BMI, Body Mass Index.
